# The ethical case for global measles eradication—justice and the Rule of Rescue

**DOI:** 10.1093/inthealth/ihaa038

**Published:** 2020-07-08

**Authors:** David N Durrheim, Jon K Andrus

**Affiliations:** School of Medicine and Public Health, University of Newcastle, Newcastle, New South Wales, Australia; Centre for Global Health, University of Colorado, Denver, Colorado, United State of America

**Keywords:** ethics, eradication, justice, measles, rescue

## Abstract

Measles causes a substantial disease burden for all countries, while mortality is greatest in underserved, marginalized populations. Global measles eradication is feasible and the strategies critically rely upon well-functioning national immunisation programs and surveillance systems. All six regions of the World Health Organisation have adopted measles elimination targets. The Rule of Rescue and the principle of justice leave no ethical place for health programs, governments, global public health bodies or donors to hide if they impede efforts to eradicate measles globally by not taking all necessary actions to establish a global eradication target and committing the resources essential to achieve this goal.

## Introduction

Measles is estimated to have claimed the lives of >142 000 people, mainly young children, in 2018; a staggering 18 000 more deaths than in 2017.^1^ The global resurgence in measles cases that began in 2016 continues unabated, with more reported cases in 2019 than the total for 2017 and 2018.^2^ The region of the Americas, due to reestablishment of endemic measles circulation in Venezuela and Brazil, and countries in Europe (Albania, Czech Republic, Greece and the UK) and the Western Pacific region (Mongolia) that had previously been verified to have interrupted measles transmission have lost their verification status.^3–5^ As a result, the argument has been made that the current global measles situation meets the criteria of a Public Health Emergency of International Concern (PHEIC), but the World Health Organization (WHO) has not declared a PHEIC.^6^ Viewed through the Rule of Rescue lens and when weighed on the scales of justice, this performance by humankind is found to be ethically wanting.

## Is measles eradication possible?

‘Measles can and should be eradicated’ was the conclusion of the Strategic Advisory Group of Experts (SAGE) Working Group on Immunization to the WHO following an exhaustive review of biological, technical, economic and programmatic evidence.^7^ Furthermore, SAGE recommended that a goal for measles eradication (reduction of global measles incidence to zero as a result of deliberate efforts) should be established.

This belief and commitment was reflected in the Global Vaccine Action Plan, which was endorsed by the World Health Assembly in 2012, setting the goal for measles elimination in five regions by 2020.^8^ Regional enthusiasm exceeded this target, with each of the six regions committing to eliminating endemic measles on or before 2020.

Measles can be eradicated; the Americas achieved zero endemic measles cases in November 2002.^9^ To that end, all regions have committed to achieving the public health goal of measles elimination, yet an unacceptable number of children died of this totally preventable disease in 2018. The worldwide epidemiological situation continues to deteriorate. Measles resurgence reflects stagnating global coverage with measles-containing vaccine (MCV), which has plateaued for the past decade at approximately 85%, about 10% lower than necessary to prevent outbreaks and cease transmission.

Fortunately, strengthening the essential immunisation program is, and should be, the foundation for eradicating measles. The key to sustainable measles elimination is investing in the primary health structure at the point of service delivery, strengthening surveillance and ensuring the necessary community engagement and political commitment to reach every child with MCV and other integrated immunisation and primary healthcare services.

The coronavirus disease 2019 pandemic is having a negative impact on routine immunization coverage in many countries and this burden is likely to fall more heavily on developing countries. Given this negative impact, we should expect to see expanded measles outbreaks in the near future. If a safe and effective pandemic vaccine becomes available, it will be crucial to piggyback routine immunization catch-up onto pandemic outreach immunization activities in an attempt to close the inequitable immunization gap between stronger and weaker health systems, while also addressing an inevitable measles resurgence.

## Ethical arguments for and against measles eradication

There is little doubt that eradication will take a major coordinated effort, but the benefits will extend well beyond extinction of the virus. To achieve the phenomenal vaccine coverage required to eradicate measles will require that health systems perform well, reach every community and protect almost every child. Thus previous ethical justification for achieving measles eradication has focused predominantly on equity.^10^ The occurrence of measles highlights inequity in health service provision, graphically illustrated by an analysis of 123 million neonatal, infant and child deaths between 2000 and 2017.^11^ The obvious clustering of deaths by low socio-economic status at a subnational scale in 99 countries mirrors the occurrence of measles. The children who are at greatest risk of severe disease have poor nutrition, co-infections and limited access to healthcare. Reaching them with immunisation will have a real effect on health inequities. As the burden of premature deaths falls disproportionately on the very young, if the global community does not respond with solidarity and commitment, the young will continue to be swindled of their ‘normal’ healthy lifespan.^12^ Because measles elimination relies so heavily on essential immunization services, reaching these children will also give them the benefit of other lifesaving vaccines.

The goal of measles eradication has been ethically questioned, with a primary concern being the attendant cost and reliability of achieving eradication. It has been argued that after achieving eradication, prophylaxis and vigilance would be replaced with indifference and trust.^13^ With the dual threats of bioterrorism in immune-naïve populations and the possibility of virus escape from a laboratory or undetected virus in marginalised communities, it is contended that embarking on measles eradication is unethical.

The principle of beneficence, and more particularly the rule of rescue, demands that those who are able, in this case governments and international donors, rescue identifiable individuals facing avoidable death, if personal sacrifice is not excessive.^14^ An analogy may be useful. We are fortunate to be surviving in a life boat. If we reach out to save a drowning victim and hoist him/her on board, will we capsize the lifeboat and risk our own lives? Given the compelling scientific evidence of the feasibility of eradication and the preventable death toll exacted annually by measles, the rule of rescue is a potent ethical sabre for compelling accelerated global action. Although the Rule of Rescue has been criticised as benefitting a few at a cost to many, this utilitarian counterargument appears flawed, given the cost-effectiveness of measles vaccination and the return on investment of eliminating measles, and concurrently rubella and congenital rubella syndrome (CRS) through using a combination vaccine. As an aside, the experience in the Americas demonstrated that the combined vaccine approach provides a side benefit of CRS elimination, a result that has been substantially cost-saving and humanitarian.^15,16^ The colossal net economic gains of eradicating measles infection, primarily through direct treatment and outbreak response costs saved (>US$2 billion per year) and disability-adjusted life year (DALY) losses prevented (>15 million DALYs per year valued at >US$63 billion), could provide much needed investment in other public health programmes.^17^

Measles eradication could be further defended from a utilitarian perspective on the grounds that the commitment to rescuing the most vulnerable will increase societal well-being by reinforcing people's belief that they live in a community that places great value upon life and fairness.^18^

## When it comes to a measles eradication goal, is there any ethical place to hide?

Beneficence is influenced by the consequences of doing nothing, the feasibility of preventing serious consequences and the scale of sacrifice. On all three counts the Rule of Rescue compels us to make every reasonable effort to eradicate measles. Recently the United Nations High Commissioner for Human Rights, Michelle Bachelet, a paediatrician and former president of Chile, penned an insightful reflection on health inequities in childhood, ‘Data on child deaths are a call for justice’.^19^ She argued that hard data must be followed by action across the whole spectrum of government and society.

At the 70th World Health Assembly (WHA) in 2017, the director-general was requested to report through the Executive Board to the 73rd WHA in 2020 ‘on the epidemiological aspects and feasibility of, and potential resource requirements for, measles and rubella eradication, taking into account the assessment of the SAGE on immunization’.^20^

Unfortunately the question of setting a measles eradication goal was not debated at the WHA in Geneva in May 2020 because SAGE concluded in their feasibility assessment just prior to the WHA that: ‘Given the current global context, achieving measles eradication is not realistic without significant further effort. There is an urgent need for all countries and regions to accelerate progress towards achieving and maintaining measles and rubella elimination goals’.^21^ Governments and international health agencies do not have any escape from the ethical compulsion to accelerate the achievement of this goal without any further delay.
